# How clinicians discuss patients’ donor registrations of consent and presumed consent in donor conversations in an opt-out system: a qualitative embedded multiple-case study

**DOI:** 10.1186/s13054-023-04581-9

**Published:** 2023-07-28

**Authors:** Sanne P. C. van Oosterhout, Anneke G. van der Niet, W. Farid Abdo, Marianne Boenink, Thomas G. V. Cherpanath, Jelle L. Epker, Angela M. Kotsopoulos, Walther N. K. A. van Mook, Hans P. C. Sonneveld, Meint Volbeda, Gert Olthuis, Jelle L. P. van Gurp

**Affiliations:** 1 Department of IQ Healthcare, Radboud Institute for Health Sciences, Radboud University Medical Center, Kapittelweg 54, 6525 EP Nijmegen, The Netherlands; 2 Department of Intensive Care Medicine, Radboud University Medical Center, Nijmegen, The Netherlands; 3 Department of Intensive Care Medicine, Academic Medical Center, University of Amsterdam, Amsterdam, The Netherlands; 4 Department of Intensive Care Medicine, Erasmus Medical Center, Rotterdam, The Netherlands; 5 Department of Intensive Care, Elisabeth Tweesteden Hospital, Tilburg, The Netherlands; 6 Department of Intensive Care Medicine, Maastricht University Medical Center, Maastricht, The Netherlands; 7 Department of Intensive Care Medicine, Isala Hospital, Zwolle, The Netherlands; 8 Department of Critical Care, University of Groningen, University Medical Center, Groningen, The Netherlands

**Keywords:** Intensive care, Organ donation, Opt-out consent, Medical ethics, End-of-life decision-making, Communication, Qualitative research, Professional-family relations

## Abstract

**Background:**

The Netherlands introduced an opt-out donor system in 2020. While the default in (presumed) consent cases is donation, family involvement adds a crucial layer of influence when applying this default in clinical practice. We explored how clinicians discuss patients’ donor registrations of (presumed) consent in donor conversations in the first years of the opt-out system.

**Methods:**

A qualitative embedded multiple-case study in eight Dutch hospitals. We performed a thematic analysis based on audio recordings and direct observations of donor conversations (*n* = 15, 7 consent and 8 presumed consent) and interviews with the clinicians involved (*n* = 16).

**Results:**

Clinicians’ personal considerations, their prior experiences with the family and contextual factors in the clinicians’ profession defined their points of departure for the conversations. Four routes to discuss patients’ donor registrations were constructed. In the Consent route (A), clinicians followed patients’ explicit donation wishes. With presumed consent, increased uncertainty in interpreting the donation wish appeared and prompted clinicians to refer to “the law” as a conversation starter and verify patients’ wishes multiple times with the family. In the Presumed consent route (B), clinicians followed the law intending to effectuate donation, which was more easily achieved when families recognised and agreed with the registration. In the Consensus route (C), clinicians provided families some participation in decision-making, while in the Family consent route (D), families were given full decisional capacity to pursue optimal grief processing.

**Conclusion:**

Donor conversations in an opt-out system are a complex interplay between seemingly straightforward donor registrations and clinician-family interactions. When clinicians are left with concerns regarding patients’ consent or families’ coping, families are given a larger role in the decision. A strict uniform application of the opt-out system is unfeasible. We suggest incorporating the four previously described routes in clinical training, stimulating discussions across cases, and encouraging public conversations about donation.

**Supplementary Information:**

The online version contains supplementary material available at 10.1186/s13054-023-04581-9.

## Background

One strategy to potentially increase the number of organ and tissue donors is a policy change from an “opt-in” donor registration system to an “opt-out” system [1]. Similar to the policy changes in the United Kingdom, the Netherlands introduced an opt-out system in 2020 [1–7]. Opt-out systems differ in how they are designed and implemented [8]. A common factor in any opt-out system is the default of donation, which means that consent for donation is presumed unless an adult registered a refusal to donate. This also changes the default for patients’ families [9].

In the former Dutch opt-in system, families had to decide about donation when patients had no registration. In the current opt-out system, family consent is no longer formally necessary in (presumed) consent cases, but the new donor law does offer families the right to oppose both presumed and registered consent. The new law describes family rights more clearly than in the previous opt-in system (Table 1 and Additional file 1). In addition, the Dutch “Kwaliteitsstandaard Donatie” (Quality Standard for Donation [QSD]) was written to explain the content of the law so that it would be clear to clinicians how to approach the registered choices and what rights the patients’ families have. The overarching aim is uniform application of the law in clinical practice (Additional file 1) [10, 11].

**Table 1:** The Dutch donor law [2–4, 7, 56]

Dutch Donor Law
In 1998, the Dutch Donor Act was introduced. This law was based on a voluntary opt-in donor registration system in which organ and tissue donation only occurred with explicit consent from the deceased donor. When no decision was registered, the patients’ family members had to decide. Despite various efforts to increase the number of donors to meet the demands for transplantation, 7 million (51%) residents remained unregistered in the Donor Register [57]
As of July 2020, the opt-in system was replaced by an active donor registration (ADR) system, i.e., an opt-out system. The opt-out system changed the default of donor registration from “family decides in case of nonregistration” to “donation”. This system aims to increase donations and save multiple lives. In addition, it might relieve pressure on patients’ families who must otherwise decide on organ and tissue donation in an often challenging, emotional situation [58]. The new donor law also describes family rights more clearly than in the previous opt-in system
Four choices for donor registration exist in the opt-out system, which are the same as in the opt-in system:
1. Consent or opt-in (with or without restrictions to specific organs or tissues),
2. Refusal or opt-out,
3. Leave the decision to a designated person, and
4. Leave the decision to family members (first and second degree)
The three *novel* aspects of the law are:
1. All persons aged 18 years or older who are not yet registered in the Donor Register receive two letters in which they are asked to register their donation wishes. If they do not register, a third letter informs them about being listed in the Donor Register as having “no objection” to organ and tissue donation. In other words, when persons do not actively deregister after these reminders, they are registered with *presumed consent*, which is legally considered the same as actively registered consent. In the Netherlands, 25 percent (3.3 million people) of people are registered with presumed consent [7, 57]
2. Registration can still be challenged by families in the case of (presumed) consent to donation if families can convincingly demonstrate a credible case that the donor registrations do not correspond to the patients’ wishes. Families must inform and explain their difficulties, with no requirements with respect to form, to the health care professionals, who can invite them to explain their difficulties
3. Donation for mentally incompetent persons is also possible. As we did not include cases with mentally incompetent persons, this aspect extends beyond the scope of the present study. We will not elaborate on donation in patients younger than 18 years old either
More information (in Dutch): https://www.rijksoverheid.nl/onderwerpen/orgaandonatie-en-weefseldonatie/actieve-donorregistratie

Although applying the default of donation seems straightforward, family involvement adds more complexity to its application in clinical practice. Evidence from other opt-out countries indicates that clinicians continue to give families’ views a decisive role in donation decision-making [8]. The goal in opt-out systems is to make nonregistration equal to consent to donation. However, families overrule a presumed consent more often than an actively registered consent in clinical practice (e.g., Wales: 26% vs. 10%) [12]. In Wales, some families still thought that whether or not to donate was their decision to make, which challenged clinicians to manage families with different expectations about their role [6]. Donor practice thus seems complex: legislation on donor registration tends to be more strictly formulated than its application in clinical practice, presumably because clinicians do not wish to exclude or harm families [6, 13].

While the QSD aims to explain the content of the law for clinical practice, clinicians may experience additional complexity in the actual application of the law in clinical practice. On the one hand, donor registrations should be leading in the conversations; on the other hand, consensus and dialogue of clinicians with the families is emphasised, and families’ satisfaction with the donation decisions is likewise stressed [10]. It has been reported that clinicians can experience difficulties balancing supporting the patients’ donor registrations on the one hand and caring for the family on the other [6]. How clinicians should achieve this balance in clinical practice is not and cannot be completely specified, as every situation and family is unique. Room for clinicians’ own professional judgements remains present in clinical practice, such as how clinicians should deal with grieving families or judge families’ hesitations. To date, limited empirical research is available about the actual clinical practice of donor conversations under opt-out legislation, and it remains unclear if and how Dutch clinicians experience complexity in applying opt-out. The present study aims to describe and analyse how Dutch clinicians discuss patients’ donor registrations in donor conversations in the first two years of the opt-out system.

## Methods

### Design and setting

The current study is part of a four-year qualitative embedded multiple-case study evaluating the implementation of the law by comparing Dutch clinical practice before and after the system change in 2020 [14]. Cases included audio recordings, and nonparticipant direct observations of donor conversations when possible, and supplementary in-depth interviews with intensive care unit (ICU) clinicians (residents, fellows and attendings), a physician assistant, nurses, and family members [14–20]. In total, 29 cases were obtained consisting of 29 conversations, 58 interviews with health care professionals (33 clinicians, 23 ICU nurses and 2 organ donor coordinators [ODCs]) and 19 with family members. In the Netherlands, ODCs are generally involved after the donor conversation, but clinicians may request their participation in the conversation based on ODCs’ donation expertise. Data collection was performed between February 2021 and December 2022 at ICUs of eight hospitals, including six tertiary university medical centres and two teaching hospitals. These hospitals were selected based on geographical spread and volume of yearly initiated organ donation procedures  [21]. In this study, we conducted a modified case study in which we selected seven consent and eight presumed consent cases (*n* = 15) with supplementary interviews with clinicians that occurred under the opt-out system.

### Case inclusion

Initially, convenience sampling was applied because the occurrence of donor conversations was unpredictable [22–24]. Cases were included in which at least donation after brain death and/or after circulatory death was discussed with the family. Cases in which only tissue donation was discussed were excluded. After six cases, purposeful sampling with maximum variation was used based on donor registration, hospital and case description. Finally, theoretical sampling was employed [22–25].

### Data collection

Observations focused mainly on interaction and nonverbal communication (e.g., eye contact, facial expressions, posture) and guided the researcher upon which elements to emphasise in the interviews. Audio recorders were provided to hospitals in case the researcher could not be present. All conversations were transcribed verbatim. Field notes were made directly after every case [26].

Interviews with clinicians explored personal experiences and perspectives and were scheduled ideally within two weeks after the donor conversation. An interview guide was developed (Additional file 1). Participants were offered the option of face-to-face, telephone or video interviews. All interviews were audio recorded and transcribed verbatim. As a member check, the researcher sent interview summaries to the clinicians and offered the option to (re)view the interview transcripts [17, 25]. All summaries were approved. Demographic and medical information were collected via Castor EDC [27].

### Data analysis

Data collection and analysis were conducted through an iterative process that entailed continuous reflection on collected data and data saturation. Emergent ideas were used to refocus the interview guide. Data collection finished at data saturation [28]: when no novel theoretical information emerged from the data.

**Fig. 1:**
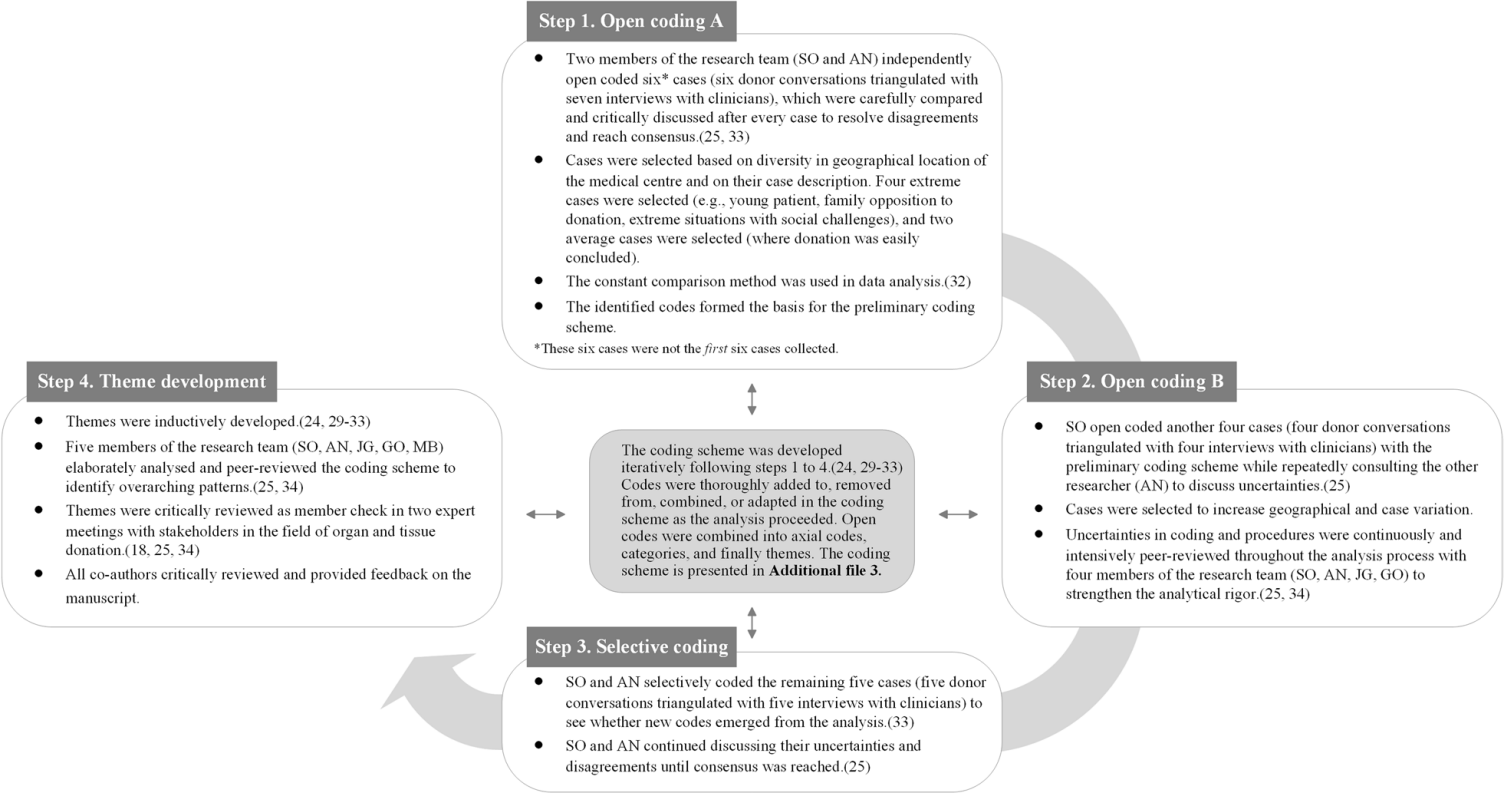
The analysis process

We used a cross-case analysis: a thematic analysis across the cases with use of the constant comparison method [24, 29–34]. Figure 1 shows the four analysis steps. Computer-assisted qualitative data analysis software facilitated the analyses, and standard descriptive statistics were used using IBM SPSS Statistics (version 27). We aimed for general theoretical insights in our results [15] and used the Consolidated Criteria for Reporting Qualitative Research for reporting (Additional file 1) [35].

## Results

**Fig. 2:**
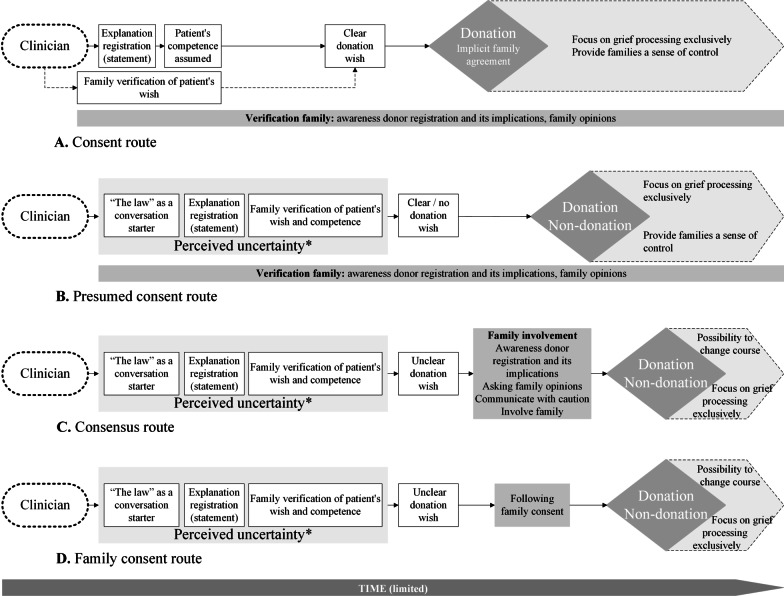
Routes of discussing patients’ donor registrations in donor conversations. The routes were not mutually exclusive, and clinicians varied between routes during the conversations. For example, elements of family involvement (Route **C**) were also present in Routes **A**, **B**, or **D**. Moreover, the routes were not linear processes in time: elements within the routes occurred at different times in the conversations. Here, our aim is to merely present the routes that clinicians applied in response to patients’ donor registrations. These do not present causality of whether a route results in donation or not. Therefore, such conclusions cannot be drawn based on these routes. *Perceived uncertainty: Compared to an actively registered consent (Route **A**), clinicians expressed more concerns about whether a donor registration of presumed consent represented an actual consent to donation. This entailed more uncertainty in interpreting the donation wish of the potential donor

We present the importance of clinicians’ points of departure and, based on our data, constructed four routes in which clinicians discuss patients’ donor registrations (Fig. 2). Table 2 shows the characteristics of the fifteen cases (Additional file 1). Donor conversations lasted from 10 to 55 min (median: 19 min). All sixteen clinicians participated in the interviews (median: 52 min; 31–61 min). Illustrative quotes are presented in Table 3. While in all cases both organs *and* tissues could be donated, clinicians considered tissue donation to be a lower priority. This resulted from the perceived additional emotional burden for the family and the additional organisational burden experienced by the clinicians themselves (Q1).

**Table 2:** Characteristics of cases of family donor conversations, patients and clinicians in an opt-out system

Case (ID)^a^	Family donor conversations (*n* = 15)				Patients (*n* = 15)				Clinicians (*n* = 16)	
	Moment during the day^b^	Decoupled^c^	Duration (min)^d^	Other attendees	Sex/age	Critical injury	Length of stay (days)	Type of donation discussed (result)	Profession	Sex/age/years of donation experience (years of ICU experience)
*Consent cases*										
14	D	Yes	20	ICU nurse, two daughters, son, two sons-in-law	F 57	ICH	7	DBD, DCD, tissue (DCD)	Donation intensivist^e^	M 55 27(15)
16	B	Yes	16	ICU nurse, spouse/partner, friend	M 63	Not known (PE)	1	DBD, DCD, tissue (DBD, tissue)	Donation intensivist	M 55 27(15)
21	C	Yes	19	ICU nurse, medical intern, son, friend	F 60	ICH	4	DCD, tissue (DCD, tissue)	Fellow	M 31 7.25(3)
26	A	Yes	22	ICU nurse in training, medical intern, spouse/partner, sister, brother-in-law	M 60	MI	14	DBD, DCD (DCD)	Intensivist	M 40 10(5)
27	B	No	19^f^	Resident, ICU nurse, spouse/partner, two daughters, two sons, brother-in-law	M 64	ICH	1	DBD, DCD, tissue (DCD)	Intensivist	M 38 6(6)
29	D	Yes	30	Resident not in training, ICU nurse, spouse/partner, two sons	M 71	ICH & CA	0.1	DBD, DCD (no donation^g^)	Fellow	M 32 5(1)
29*	A	No	32	ICU nurse, psychiatrist, mother, father	F 25	CA	2	DBD, DCD, tissue (DBD)	Fellow	F 37 6(12)
*Presumed consent cases*										
4*	D	Yes	1:15 2:14	ICU nurse, two sons, daughter-in-law, sister-in-law	M 62	TBI, HBI	1	DBD, DCD (no donation^h^)	Donation intensivist	F 48 16(16)
8*	B	No	39	ICU nurse, neurosurgeon, spouse/partner, daughter	M 72	TBI	1	DCD, tissue (no donation^g^)	Intensivist	F 58 25(25)
11*	A	No	1:35 2:21	ICU nurse in training, fellow, spouse/partner, son, daughter	M 68	ICH	0.5	DBD, DCD (DCD)	Intensivist	M 44 11(11)
15	B	No	19	ICU nurse, daughter with her boyfriend, mother	F 60	ICH	1	DBD (DBD)	Fellow	M 31 7(2.75)
18	C	Yes	12	ICU nurse, ICU nurse in training, spouse/partner, son, daughter, daughter-in-law, son-in-law, brother, sister-in-law	M 53	ICH	0.3	DBD, tissue (no donation^g^)	Intensivist	M 62 31(31)
19	B	Yes	10	ICU nurse, ICU nurse in training, ODC, spouse/partner, mother, mother-in-law	F 41	CVA	1.5	** (no donation^h^)	Fellow Intensivist	F 30 2.5(2.5) M 47 22(16)
20	B	Yes	14	ICU nurse, fellow, two sisters, daughter, niece	M 61	CVA	2	DBD, DCD, tissue (DCD)	ICU resident not in training	F 32 1.75(1.75)
23	B	Yes	18	ICU nurse, medical intern, spouse/partner, daughter, son-in-law	M 63	TBI	4	DCD (DCD)	Donation intensivist	M 43 17(10)

*DCD* donation after circulatory death, *DBD* donation after brain death, *CA* cardiac arrest, *CVA* cerebral vascular accident, *ICH* intracranial haemorrhage, *ICU* intensive care unit, *HBI* hypoxic brain injury, *MI* myocardial infarction, *ODC* organ donor coordinator, *PE* pulmonary embolism, *TBI* traumatic brain injury

^a^We retained the original case numbers. Therefore, cases are not numbered from 1 to 15, as we only selected the consent and presumed consent cases

^b^Moment during the day: A: Morning, B: Afternoon, C: Evening, D: Night

^c^From the bad news conversation

^d^Rounded to minutes. When the donor conversation was not decoupled from the bad news conversation, the duration includes the duration of the bad news conversation and the donor conversation together. The donor conversations of cases 8 and 10 were separated into two conversations

^e^A donation intensivist is an intensivist with a specific focus on donation

^f^The conversation was not entirely recorded due to low battery level of the audio recorder

^g^Initiated procedure, but no donation because organs and, if applicable, tissues were not usable/rejected, or the patient did not die within two hours (for DCD)

^h^No initiated procedure due to family opposition in the donor conversation (not patient’s wish or family’s potential psychological harm)

*The researcher directly observed and audio recorded the donor conversation

**No information about donation: only mentioning the option for organ and tissue donation

**Table 3:** Illustrative quotations

Q. no.	ID number	Quote
*Results*		
1	I11Case011	I, and with me probably many other clinicians, pay far too little attention to it [tissue donation] and have limited knowledge about it. It is an addition [to organ donation]: you don’t want to overload the family either. You’re satisfied with what you may have achieved [organ donation] and at some moment it is enough for yourself or the family
*Clinicians’ points of departure for a donor conversation*		
2	I30Case029	I’m positive about it [donation]. (..) Yes, I think it does [influencing professional conduct]. I do think that it [donation] is easier to convey or to get people motivated for when you can show the usefulness of it
3	I20Case019	Maybe also because you have a bit of internal resistance to impose it [donation in the case of presumed consent] on them [the family]. I want to be compliant with the law, which is of course one of the arguments why the conversation is going the way it is. So, you can’t avoid it. However, it’s a bit uncomfortable. That you go from something so emotional, from someone’s grief to something very formal, namely, a legal framework on which we base our argumentation structure
4	I15Case014	It was no surprise what was registered in the Donor Register [in this case: consent]. They immediately said: ‘yes, yes, yes’, they understood. So, in that sense, it was relatively easy. We had a common base on which you can continue to build on. (..) No [There were no disagreements with the family] and not within the family either
5	I15Case014	It’s what I said about finding a ‘landing site’ [for donation with the family], that’s the most crucial thing: that you [clinician] find an entry point. In addition, as long as the entry point isn’t there, there is no acceptance that things are truly radically different from now on and that someone is being ripped out of their [families] lives. Of course, this applies to a greater extent to younger patients and more unexpected deaths. If that acceptance isn’t there, you can’t truly have a conversation about donation. (..) So people must be convinced [about the imminent death]
6	I17Case015	However, if you can already estimate for yourself, okay (..) this truly is not going to be a donation, but you still have to check that with the family, that you do notice that you are going to look for arguments within yourself on which you actually hold back the donation. So that you actually no longer have to ask the question or that you can just keep it [the conversation] very short (..) When prior communication with the family has been difficult, then you think: poah, it’s going to be very complicated if I must ask the donation question in a very open manner. (..) Sometimes, it can help to check for yourself: are there any other reasons…? (..) that you (..) look even stricter at arguments whether it [donation] is possible at all or whether there are simply already contra-indications not to do it [pursue the donation]. (..) It perhaps tends faster toward those contra-indications than you would normally do
7	I21Case019	You must think a lot about the legislation, what the consent… or how patient is registered and what it then means, right? As a doctor you must be almost half a lawyer to tell it all apart. That makes it a bit difficult. That [presumed consent] is now clear, now that I’ve done it once [the donor conversation], but I must think every time which 5 or 4 forms of registration exist
8	I11Case011	I talked to [name donation intensivist*] about it, because it was a person who was registered with ‘no objection’ [presumed consent] and [name donation intensivist] (..), of course, considers it important how we use and apply that. (..) [name donation intensivist] emphasised the approach of the new donor law [laughs]: that ‘no objection’ is not ‘we ask family consent’, but that there *is* consent, that in particular actually. That you take that into account. So don’t pretend that there was no [donor] registration, but that we consider it as consent
9	I20Case019	We already felt it coming [resistance], because the ICU nurse indicated that she truly sensed resistance to donation. In addition, yes, our approach since the new donor law is that (..) we would like to start the donation after all. (..) Therefore, we thought, we need slightly more context and some sort of subtitles for the donation from the organ donor coordinator. Therefore, we asked her to join us. (..) I think she certainly added value in getting them [the family] to think a bit [about donation]
10	I4Case004	With such a complicated case in the middle of the night, (..) it takes an awful lot of time, and we have *number* other patients too of course. Therefore, you can’t just take infinite time for it [discussing donation]. (..). Look, you take the time, and you make time for it, but of course you can’t go on endlessly about it, of course you also have other patients who you must give your attention to, those who actually still have a chance of survival. (..) During one of the conversations, I had to leave for CPR [cardiopulmonary resuscitation], and then you must [remind] yourself all over again: oh yes, where was I? Oh yes, where were we?
*Routes of discussing patients’ donor registrations in donor conversations*		
11	I26Case026	It is a constant search for a balance between, say, the donation process and the grieving process, so you also must plead for both. (..) You always must make sure it doesn’t go off the rails on one of those fronts. Therefore, I think that going along too easily with the wishes of the family, while something else is registered, I would call that 'derailing'. However, on the other hand, it is also … If continuing the donation, yes, is hurtful toward the family, if there is a breach of trust, that is also derailed. You must prevent that. (..) And to say what is more important: donation or a good contact with a good grieving process. (..) I think that also differs a bit from moment to moment. (..) It is a very crazy comparison if you must draw up a balance of motives and arguments between the interests of one individual [the patient]—possibly with the family around—on the one hand, and the social interest of the organ donation on the other
12	I15Case014	Then [in consent cases], you often notice that the interests of organ donation take precedence, because that path is already being taken [due to the positive donor registration]. In addition, then you must make sure that you keep the family on track. Intensive guidance and… However, also giving direction—That is what we also do. When someone [health care professional] says ‘but we can wait maybe another 6 h [for diagnosing brain death]’, that you [clinician] then say ‘no’. Our job then is to protect the family from being too utilitarian—which just no longer suits their emotional capacity. (..) You must coach the family. So also during the course [of donation], you constantly have to keep in touch with the family, the guidance of ‘where are you [in the process]?’ To check again: is it [the procedure] bearable for you? Or what can we do differently to make it better for you?
Consent cases *Route A: Consent*		
13	Case014	Clinician: We would like to follow up on her wish, the wish [consent] she indicated herself in the donor register. (…) And she made her wish very clear, and therefore, we [the health care professionals] want to fulfil that wish as best as possible
14	Case014	Clinician: (..) For this, we always consult the Donor Register, and she’s in it. She was registered in it [with consent]. Maybe you know that too? Daughter 1: Yes (..) Clinician: (..) We would like to follow-up on her wish, what she herself has indicated in the Register Daughter 2: Please Clinician: Do you agree with that? Daughter 1: Definitely Son: Yes, good
15	I26Case026	There are some comments that indicate that they [the family] are okay with it [the donation]. Sometimes they say, ‘we know that it is his own choice’ or (…) ‘nevertheless, his death can lead to something positive’. (…) When amongst the people present no discussion about donation rises, and they simultaneously nod as a response to donation, then I assume a somewhat alleged permission… or alleged agreement of the family to at least further initiate the donation process. (…) I don’t remember exactly what they said in this case, but I think it was after one or two sentences, that I got the impression: well, donation is all right
16	I28Case027	To give the family the feeling that they had some control, so to speak. (..) This sense of control of the family is especially that you [as clinician] emphasise that everything is possible. (..) And you give a few examples of that [ “everything”]. That if they want to be with their loved one all the time through the donation procedure, that’s fine. That if they want to leave [the hospital] and just want to be called [by telephone], that’s fine. That if the partner would like to lay in bed with the patient, that’s fine. However, that it [the donation procedure] should be bearable, it must be bearable [for the family]
17	Case028	Clinician: The most important message I want to give you is that a lot [regarding donation] is possible. There’s – I think almost anything is possible, but just try to keep in touch with us [health care professionals], and then we’ll see what’s feasible and how we can support you. (..) It’s a tough time
Presumed consent cases		
18	I7Case008	I would say that apparently it is not their last will [in case of presumed consent], but it is not so clear, is it? Last will is: ‘yes or no’. However, not reacting and being obliged to it [donation], I don’t know if that is truly a last will
19	I20Case019	And I find that second part [that presumed consent equals ‘donation’] very difficult. In this donor conversation, there was clearly immediate resistance [to donation, after introducing the topic] and to bring a counterbalance then… to truly slow people down in their reflections that they must choose a course at that moment. I find that very difficult, because you have the feeling that you have to bypass the family a bit in their wishes. Therefore, in that sense it is not always a very pleasant announcement
20	Case011	Clinician: Tell me a little bit, what did he say, what did you talk about [regarding donation]? (..) C: However, he said, if I understand correctly, if there is anything I can help someone with… than he would like that (..) C: I especially want to hear a little bit more from you… that’s what you tell ma’am, that he basically supported that [donation] (..) C: Therefore, what I’m taking from you now is that what actually is registered now [presumed consent], even though he didn’t actively do that, but just by doing nothing- (..) C: That [presumed consent] suits him? (..) C: Okay, but still you guys talked about it and does that fit with what- (..) C: What he thinks, and for you, it suits him and it’s not a problem for you either
21	Case011	Clinician: The rules that are now formulated for him, will be literally: it [donation] is his last will that he wants in principle, because he has not opposed to it (..) (..) Clinician: If you as a family do not make it clear that he or you have important disagreements to this for certain reasons, then we will of course just want to follow his last will. That is how the legislator wants it (..)
*Route B: Presumed consent*		
22	Case023	Clinician: (..) He is registered with ‘presumed consent’ Partner: That’s right C: Yes, did you know that? P: Yes C: Did he consciously bring that about? P: Yes C: Yes, knowing that that actually means that you give consent to organ donation P: YES C: Yes, that is also the meaning it [presumed consent] has. In addition, you talked about that together at that time, of what you thought about his point of view or…? P: Yes, we talked about that too C: Yes, and what do you think of his point of view? P: Well, he had to know that himself. It’s his decision
23	I20Case019	It’s not some kind of shared decision-making, such as offering a treatment in the outpatient clinic (..) There’s just a law, that we’re supposed to abide or try to abide. (..) It’s a decision that we [made] from a legal point of view in consultation with the [medical] team (..). Then, it will be a conversation with the family where you want to announce what we are planning to do [donation] (..)
24	Case015	In principle, the approach of the hospital is (..) to cooperate with it, organ donation. (..) So that we are going to initiate everything to be able to donate organs to other people
25	Casus019	Clinician (intensivist): If you are convinced [that donation was not their known wish of the patient] and it does sound that way, it is clear to me that that [donation] would be against her will if she could still have a say on this (spouse: yes well…), then we just shouldn’t do it, I think Spouse: I think that’s the wisest thing to do Clinician (fellow): I think we agree on that too, right?
26	I7Case008	There will always be parts [families] that say ‘yes’ and some that say ‘no’ [to donation]. Then, you should still try to explain how the registration arose, that it is the law. (..) Just [emphasise] that we can also do a lot of good for other people [with donation]. To try to persuade them anyway
*Route C: Consensus with the family and family involvement*		
27	I17Case015	Therefore, it’s not my intention to truly ask consent, but I do think that it also feels much better for uhm… everyone, if they [the family] have had the feeling that there is still some kind of participation or whether it is a joint decision. Even if it is not a joint decision [by law]
28	I20Case019	I think in that moment [when introducing donation], you just have to provide a stage for their [family’s] feelings so that they don’t feel completely unheard or that you have a huge battle with the family, but after that, you also have to slow them down a bit in the sense that yeah ‘it may feel like a question [for consent], but in fact it isn’t. However, completely disconnection them [the family] from that decision is not truly possible either
29	I20Case019	In case of presumed consent, (..) you don’t know it for sure [whether the patient gives consent for donation], so that’s why I also let the opinion of the family weigh more strongly, I guess
30	I26Case026	Then, I think in clinical practice – even though I don’t think the law is meant in that way – it [donation] will often fail because we want to keep a somewhat good contact with the family; at least not a total break of contact. (..) However, if, despite my efforts in which we show that we are serious about it [the donation] the situation remains that they [the family] continue to oppose to the donation, then yeah, they will eventually get what they want, so to speak
31	I15Case014	The family keeps a vote in it [whether donation is pursued] and is still leading in what happens in the end. Otherwise, you distort the relationships you’ve built. For such a delicate subject. (..) Well, if it truly turns into a very severe conflict then I’m not going to persist. Then, it may be that the one who has difficulties with the donation gets the heaviest vote. However, that must be discussed intensively with them: why do you come to that decision? What’s holding you back?
32	I22Case020	If they [the family] then say ‘he truly didn’t want that [donation]’ or for whatever reasons, then I’d be quicker to accept that if someone is registered with presumed consent [compared to an actively registered consent]. Instead of… because with consent I can say ‘yes, but he explicitly indicated that [consent] himself’. If there is presumed consent, yes, I think that the opinion of the family weighs more heavily for me
33	I29Case028	The goal of every donor conversation is to effectuate donation, but yeah, somewhere in your head, (..) there is a kind of dual advocate. Who on the one hand wants to create as much health as possible and therefore harvest as many organs as possible and give them to other people, but on the other hand, there is also someone who must tell the family that a family member is dying, and you also want to offer those people some form of support. I do find it more difficult to convince people that [donation] is what the patient would have wanted if people [the family], who know the patient much better, certainly claim the opposite
34	Case011	Clinician: (..) I think what’s important is that we align it [the donation procedures] with ma’ams [spouse] needs and your [daughter and son] needs. Taking that together with what he [the patient] had wanted [donation] and that we try to bring those things together as best we can, and sometimes it can’t be perfect and somewhere it’s too much at some point and then you have to stop it [donation] too
*Route D: Family consent*		
35	Case004	Clinician: If you do not respond to the letters [with the request to register donation wishes], then you actually agree with donation, but even then, we always want to ask it to you, as a family, because we think that’s important Sister-in-law: So, they [the adult children of the patient] can still say ‘we don’t want to pursue the donation’ or ‘we do want to pursue the donation’? Can they decide that as relatives? Clinician: yes, that’s indeed possible
36	I4Case004	Look, those people were totally shaken and confused too, and young too, there’s a lot coming at them. (..) Therefore, yeah, I didn’t think you had to go on about that [that it should be a donation based on the presumed consent registration] for a very long time. We gave those people a lot of time to think about it [donation], I thought that was more important than putting forwards the arguments why they thought that he shouldn’t be a donor after all. Because we just asked the donation question [for consent]

*A donation intensivist is an intensivist with a specific focus on donation

### Clinicians’ points of departure for a donor conversation

Three general aspects defined the clinicians’ points of departure. First, clinicians brought their personal considerations and preferences to the conversations. Clinicians had various opinions about donation and the new donor law, varying from clinicians who were aware of the potential influence of their personal donation attitude (Q2) to clinicians who consciously separated personal opinions from their professional attitude. Hesitation was expressed concerning living up to (part of) the new law, which made (parts of) the donor conversations challenging (Q3).

Second, clinicians’ prior experiences with the family and how clinicians judge family members’ characters were considered relevant. Clinicians felt more comfortable introducing donation when they were positive about prior contact moments and had the feeling of being able to anticipate the families’ responses or when the family had already conveyed a constructive and positive attitude about donation during the ICU stay (Q4). Conversations were in general more comfortable for clinicians when they knew that the families had already accepted the poor prognoses of their relatives and were thus more receptive discussing subsequent steps such as donation (Q5). A clinician mentioned that when he expected a strong hesitancy toward donation from the family, he tended to frame the conversation towards nondonation (Q6).

Third, contextual factors in the clinicians’ profession were relevant. Clinicians expressed a lack of experience with applying the new law in particular or with donor conversations in general, which resulted in a lack of robust knowledge (Q7). If well prepared, for example, through discussions with colleagues, clinicians became more aware of how to notify the donation default according to the law (Q8). Colleagues (for example, donation intensivists, i.e., an intensivist with a specific focus on donation, ICU nurses or ODCs) participating in the donor conversations were valued because they added expert information and supported performing the donor conversation according to the law (Q9). Clinicians noted that the busy ICU environment influenced the flow of donor conversations due to potential interruptions and the need to reach a conclusion on whether or not to initiate a donation procedure (Q10).

### Routes of discussing patients’ donor registrations in donor conversations

Figure 2 shows four nonmutually exclusive routes in which clinicians discussed patients’ donor registrations. The routes were not linear processes in time during conversations: elements within the routes occurred at different times, and clinicians varied between routes. In all routes, clinicians perceived themselves to continuously balance five goals, which were given variable weight based on clinicians’ points of departure and the course of the conversation (Q11):

1. Abide by the new donor law;
2. Fulfil patient’s donation wish;
3. Reach consensus with the family and avoid conflicts;
4. Enable optimal grieving for the family;
5. Retrieve organs and tissues for the recipients.

Guiding and comforting the family was central for clinicians, since the family was the clinicians’ conversational partner and had to cope with the loss and donation (Q12).

#### Consent cases

##### Route A: Consent

Clinicians in this route stated consent registration as a clear donation wish of the patient, and that donation would therefore be initiated (Q13) (Fig. 2A). They assumed that these patients were mentally competent when they registered their wish, as there was no reason to assume differently based upon the medical history and talks with the family. Clinicians acted as representatives of the patients and felt comfortable with these conversations aiming to fulfil the patients’ donation wishes (goal 2).

All consent cases herein led to an initiated donation procedure. Family involvement included verifying whether families were aware of the registration and its implications, patients’ wishes, and family members’ personal opinions about donation (Q14). Apart from explicit family reactions, implicit family reactions were sufficient to assume agreement and pursue donation-related information (Q15).

When donation was confirmed, clinicians paid exclusive attention to the family’s grief (goal 4) and sought to provide the family with a sense of control in the procedure from this moment on while taking into account each family’s emotional capacity (Q16). For instance, families could exclude organs or tissues from donation or help decide on the type of donation and timing (Q17). Clinicians affirmed going through the donation process collectively.

#### Presumed consent cases

Many clinicians expressed concerns and uncertainties regarding whether presumed consent represented the patient’s actual consent to donate (Q18). They stated that laxity and misunderstandings about presumed consent implications could also have resulted in such registrations. A clinician felt uncomfortable introducing presumed consent and thus confirming permission for donation, especially when families believed they had a choice (Q19). Clinicians used a variety of expressions in the conversations to refer to “presumed consent”, such as a donation wish, permission for donation or no objection to donation.

Clinicians reported two ways in particular to cope with their concerns. First, due to the increased uncertainty, clinicians tried to verify patients’ donation wishes with the families, often multiple times, in contrast with patients with actively registered consent (Q20). Second, clinicians referred to “the law” as a conversation starter (Q21), indicating that they were legally obligated to discuss donation, and emphasised their task as “messenger” and their impartiality in the situation. Thus, the law supported clinicians in introducing donation, which was occasionally followed by emphasising the potential positive donation results (goal 5).

##### Route B: Presumed consent

When families confirmed that the presumed consent registration was indeed representing a donation wish, pursuing donation was easier for clinicians (Fig. 2B). Route B was present when families recognised or agreed with the registration and, particularly, its implications (Q22). Clinicians in this route seemed to strictly follow and express the law (goal 1) (Q23). When families challenged or questioned aspects of donation, such as “do not touch the body of my loved one”, clinicians emphasised patients’ donation wishes (goal 2), the law’s implications, their individual and broader support for these implications (Q24), or agreed with families who provided credible evidence that donation was not aligned with the patients’ wishes (Q25). We observed clinicians valuing and expressing the potential positive donation results (goal 5), and they were inclined to devote some effort to persuade families to initiate the donation (Q26).

##### Route C: Consensus

In this route, clinicians intended to provide the family with a sense of participation in decision-making (Q27) (Fig. 2C), which was experienced as inevitable (Q28). This occurred when family verification did not provide clinicians with a clear conclusion about the patients’ donation wishes (Q29). In this route, families seemed either unaware of the presumed consent registration or additionally did not even know the donation wish of their relative. Compared to Routes A and B, clinicians took ample time to ask the family if they were aware of the registration and its implications, about their own donation opinions, and provided time for the family to contemplate donation and whether it followed their relative’s point of view.

Clinicians in this route communicated about the potential donation (goal 1) with caution. The goal of reaching consensus and avoiding conflicts with the family (goal 3) had substantial weight in this route (Q30). However, goal 3 was adhered to in all routes, as clinicians reported that families were given the final say to prevent breaches of trust and complaints if families continued to express resistance to donation (Q31).

Clinicians variously managed family resistance or questions about donation. They indicated that exploring the reasons for resistance was essential, provided the family more time or additional information about the donation, or anticipated family structures and dynamics. Clinicians were inclined to approve family oppositions more easily in presumed consent cases in contrast to consent cases, where oppositions needed to be well founded (Q32). According to clinicians, initial hesitancy to donate was difficult to reverse, and while some steering to donation was allowed, persuading or counterbalancing was unwanted and considered counterproductive (Q33). They did not pursue donation when families provided valid reasons for nondonation, such as when goals 2, 3 or 4 were violated. Nondonation conflicted with goal 5, which became more relevant with high donor potential (e.g., young patient).

Finally, and in contrast to Route A, clinicians framed possibilities to withdraw from the initiated donation procedure if families’ emotional capacity (goal 4) would be exceeded (Q34).

##### Route D: Family consent

The clinicians herein actively provided room for the families to decide about donation (Q35) (Fig. 2D). These families were given decisional power about donation in a way similar to donor conversations in which the patient had a “family consent” registration or no registration as part of the former opt-in system. In other cases, clinicians acknowledged that families had no decisional power but still used language expressions, often unconsciously, suggesting this, such as “question/answer” or “decide/choice”. Families also questioned whether they had decisional rights. Route D was particularly present when clinicians viewed the case as complicated (e.g., young patient), and families experienced difficulties in coping with the situation. Clinicians experienced that the new law (goal 1) did not suit these situations, and they emphasised optimal grief processing and family guidance (goal 4) (Q36). Compared with other routes, clinicians provided the family more time to process the situation, leading to longer or multiple conversations (Table 2).

## Discussion

This is the first study to explore how clinicians discuss patients’ donor registrations of (presumed) consent in donor conversations in an opt-out system. Four routes were identified (Fig. 2), in which clinicians’ personal considerations, their prior experiences with the family, and contextual factors in the clinicians’ profession defined their points of departure. We showed that clinicians perceived that they continuously balanced five goals defending the interests of the patient (goal 2) and the family (goals 3 and 4) and aiming to conscientiously apply the new donor law (goal 1) and limit transplant waiting lists for the donor recipients (goal 5).

The change to opt-out legislation was driven by rational-utilitarian considerations to help as many potential recipients of organs and tissues as possible (goal 5) (Table 1) [36]. As the default is donation, opt-out legislation makes use of nudges to affect donor registration rates, which is not a morally neutral point of departure and can affect people’s decision-making and autonomy [37–41]. The registrations as found in the donor register are leading according to the law (goal 1), and they are presented as such in Routes A and B. The law also provides families the right to overrule the donor registrations and clinicians the freedom in interpreting families’ coping and refusals in clinical practice [4, 10]. Although it is not feasible to describe the complex and dynamic interplay between families and clinicians in relation to donor registrations in the law, questions remain regarding how family influence and coping should be weighted in donation decision-making [8, 42–45].

In clinical practice, we observed that clinicians sometimes shifted from the rational-utilitarian approach emphasising donation (goal 5) to a morally neutral approach where the desired outcome is a *good process* (goals 3 and 4) with the grieving family (which may or may not include donation) [36]. Our research showed that this occurred particularly in conversations in which families were unaware of patients’ donor registrations or donation wishes or were highly emotional (Routes C and D). One explanation for clinicians’ shift to moral neutrality may be that the donation default raises challenges for them due to concerns of whether presumed consent represents actual consent. Using “the law” as a conversation starter and extensive family verification can be explained by these concerns, which seem legitimate when people are unaware of the system changes and its consequences and view the presumed consent registration as unclear and open to ambiguity [46–48]. Moreover, these concerns indicate that the formulation of a presumed consent registration as “last wish” in the conversation, as occurred in some cases in this study, might feel uncomfortable for clinicians.

Another explanation related to this may be that clinicians regularly perform the donor conversation as if it is an attempt to obtain an informed decision, such as in end-of-life conversations, or related to starting or withholding invasive treatments that can impact life and death. They highly value standards of informed consent, as this is a central norm in their professional practice [36, 44]. Normally, patients (or their surrogate decision-makers) must be adequately informed about the treatment, its potential benefits and risks, and consequences of withholding treatment; have decisional capacity; and be free of coercion or pressure [49]. Strikingly, consent cases provide clinicians with compelling evidence about patients’ informed decisions [44], while presumed consent cases leave clinicians with additional doubt about how well informed the registration is. This increases the need to verify donor registration, involve the family or treat the family as a surrogate decision-maker*.* As advocates for patients, striving for decisive conclusions about, and fulfilling, the patients’ wishes is clinicians’ first concern (goal 2) and emphasised in the law [10]. We show that prioritising optimal grief processing and avoiding conflicts (goals 3 and 4) [50] enables clinicians to have the desired outcome of a *good process*. The inclination to gain informed consent and the importance of guiding and comforting the family in all routes indicate that our results are in line with Streat’s recommendation for a morally neutral approach in clinical practice [36].

### Strengths and limitations

A strength of our study is the in-depth insight into how the Dutch opt-out system is applied in clinical practice. We present a unique and detailed report of the complexities of donor conversations and clinicians’ views, which can only be provided through this type of research. However, there are some limitations. Although we included only one case with theoretical sampling and did not succeed in including cases with ethnic minority groups despite translated information letters, we consider the findings representative of the Dutch situation, as we included hospitals nationwide and member checking acknowledged the four constructed routes. As findings may be coshaped by Dutch culture and policy, our results cannot be easily transferred to other countries. Nevertheless, countries with opt-out systems may observe similarities. Moreover, data were collected shortly after the system change and during the COVID-19 pandemic, which could have resulted in clinicians being unexperienced with and untrained for presumed consent cases. Follow-up research after several years is therefore recommended. Finally, as postmortal donation may be a sensitive topic to discuss and some time passed between the donor conversations and interviews, social desirability and recall bias cannot be precluded despite the researcher’s interview skills. Data triangulation limited these risks.

### Implications

Our findings highlight three implications for implementing opt-out donation policies. First, lawmaker’s desire of aligning donation with donor registrations is unfeasible due to the complexity of clinical situations where family conversations occur and variations in individual clinical approaches, resulting in the four different routes. We believe that clinical practice could benefit from more uniformity than currently revealed. Clinicians might be supported by frequent discussions about (morally) significant similarities and differences across cases and how they apply the law in clinical practice. This provides opportunities to build and share a morisprudence that will solidify practice [51]. 

Second, despite various strategies, such as the QSD, online education tools and communication trainings, our data show that clinicians still encounter challenges in navigating conversations related to the law. We affirmed that clinicians were mostly untrained and less experienced with presumed consent conversations. In addition, they seem unaware of how they discuss the donor registrations and the language they use, and how these factors might influence the future course [38]. To address this, we suggest incorporating cases that represent all four routes in training programmes to foster discussions across cases and potentially improve uniformity in application of the law. Specifically, training clinicians to initiate conversations using Route A or B and follow up with Route C depending on family reactions can potentially improve the (presumed) consent conversations. While suggesting family consent (Route D) must be avoided, these situations can be used for reflection and discussions on handling family grief. Moreover, discussing the distinction between obtaining informed consent in regular ICU care and in donation practice can also assist. Incorporating the four routes in trainings aids individual clinicians’ recognition and understanding of their approaches, inclinations, and their influence on outcomes, which ultimately might enhance the effectiveness of training programmes in terms of outcomes and uniformity.

Third, although public campaigns were launched before the law’s introduction [7], families, especially in cases involving Routes C and D, were unaware of the patients’ donor registrations or wishes. In the Netherlands, 60% of the people registered with presumed consent had not discussed the registration with their families [52]. This clearly complicated the situation for clinicians, as shown in our data, and probably added to clinicians’ concerns about presumed consent. It indicates the need for more creative public campaigns to encourage conversations about donation and last wishes, especially targeting groups least aware of the donor law’s changes [44, 53]. In the Netherlands, these are youth, those of low socioeconomic status, and ethnic minority groups [52]. A creative suggestion is entertainment education [54, 55] combined with increasing awareness during occasions such as passport or driving license applications, providing information about donation, including but not limited to registration options and their meanings [44].

## Conclusion

Our study shows that donor conversations in an opt-out system are a complex interplay between donor registrations that seem straightforward and clinician-family interactions. Clinicians are confronted with the challenging task of combining goals defending the interests of the potential donor and the family while also conscientiously applying the donor law and limiting transplant waiting lists for donor recipients. When clinicians remain somewhat uncertain about the patients’ wishes or are concerned about families’ coping, they turn to the patients’ families. Clinicians desire the common routine of informed consent to give shape to a good process with the grieving family. We advise training programmes to raise awareness about the four routes, to finetune their content accordingly and to encourage clinicians’ discussions across cases and conversations about donation among the public.

## Supplementary Information

**Additional file 1**. The Quality Standard for Donation. The Quality Standard for Donation is described and explained in this additional file.** Additional file 2**. Topic list for clinician interviews. In this additional file, the topic list for the clinician interviews is reported.** Additional file 3**. Coding scheme for (presumed) consent cases and supplementary interviews with clinicians. The coding scheme for (presumed) consent cases and supplementary interviews with clinicians is reported in Additional file 3.** Additional file 4**. Consolidated criteria for reporting qualitative studies (COREQ): 32-item checklist. This additional file shows the COREQ checklist. **Additional file 5**. Characteristics of cases of organ and tissue donor conversations (*n*=15), patients (*n*=15) and clinicians (*n*=16) in an opt-out system. Additional file 5 informs about the characteristics of included cases (family donor conversations, patients and clinicians) in the Dutch opt-out system.

## Data Availability

The data supporting the findings of the current study are available from the corresponding author on reasonable request.

## Abbreviations

ICU: Intensive care unit
ODC: Organ donor coordinator
QSD: Quality Standard for Donation (in Dutch: “Kwaliteitsstandaard Donatie”)
