# Supplementary material for: How clinicians discuss patients’ donor registrations of consent and presumed consent in donor conversations in an opt-out system: a qualitative embedded multiple-case study
Source: Crit Care. 2023 Jul 28;27:299. doi: 10.1186/s13054-023-04581-9 (PMC10375668; doi:10.1186/s13054-023-04581-9)
Supplement: Supplementary file 1 — Additional file 1. The Quality Standard for Donation. The Quality Standard for Donation is described and explained in this additional file. Additional file 2. Topic list for clinician interviews. In this additional file, the topic list for the clinician interviews is reported. Additional file 3. Coding scheme for (presumed) consent cases and supplementary interviews with clinicians. The coding scheme for (presumed) consent cases and supplementary interviews with clinicians is reported in Additional file 3. Additional file 4. Consolidated criteria for reporting qualitative studies (COREQ): 32-item checklist. This additional file shows the COREQ checklist. Additional file 5. Characteristics of cases of organ and tissue donor conversations (n=15), patients (n=15) and clinicians (n=16) in an opt-out system. Additional file 5 informs about the characteristics of included cases (family donor conversations, patients and clinicians) in the Dutch opt-out system. [file 13054_2023_4581_MOESM1_ESM.docx]

**SUPPLEMENTARY INFORMATION 1**

**Additional file 1.** The Quality Standard for Donation.(1-7)

| **Quality Standard for Donation**  In the Netherlands, the new donor law was succeeded by a motion that required the development of the Dutch “Kwaliteitsstandaard Donatie” (Quality Standard for Donation [QSD]), which strives for nationwide uniform care for and contact during donor conversations with the families of deceased donors. It was written to explain the new donor law to health care professionals with the aim to ensure a uniform application of the law in clinical practice. It explains the content of the law so that it would be clear to health care professionals how to approach the registered choices and what rights the patients’ families have. In other words, it should support professionals with applying opt-out legislation in clinical practice. The QSD translates the novel aspects of the Donor Act into practice and clarifies the roles of health care professionals and families. It describes how health care professionals should discuss patients’ donor registrations and donation with patients’ families according to the donor registration options. Notably, the QSD also applies to tissue donor conversations outside the intensive care unit (ICU) and is applicable to all health care professionals performing these conversations. However, within the scope of the present research, we will refer to all of them as “clinicians”.  The donor conversations entail all conversations with families in the context of a potential donation, and each donor conversation is preceded by a bad news conversation. The donor conversation consists of two parts:  1) An initial part, where the donor registration is discussed with the family, initial information about donation is given, and, when needed, family consent is requested. In case of (presumed) consent, informing the family is sufficient.  2) A specific part, where specific information about donation is given, the specific organs and tissues for donation are determined, social and medical data of the patient are asked, and the planning for the donation is discussed. This part of the conversation can also be performed by the Dutch organ donor coordinators. A strict separation between the initial and specific part does not always exist, as these can also be present in the same conversation depending on the specific information needs of the family.  To summarise, several general elements in the QSD apply to all donor conversations:   - Clinicians must always inform families about a potential donation, which is the goal of the conversation. They must make an effort to reach the family. If family members (first and second degree) are absent or unavailable, informing friends or acquaintances is sufficient in case of registered (presumed) consent. If nobody can be contacted, donation cannot be pursued. - The aim of the donor conversation is to promote family satisfaction with the donation decision. - Clinicians should act according to patients’ wishes. The donor registration is leading. - Clinicians have the obligation to verify patients’ decision-making capacities at the time of their donor registrations. - Families are given time to react, to process the information, and to ask questions. - Clinicians should treat families in a correct manner and with a proper approach where empathy, clear communication and genuine interest are important. - All conversations, and potential conflict resolution, should be conducted in dialogue with the family. When there is a conflict or the family opposes donation, clinicians should attempt to reach consensus with the family. - Clinicians should provide families with correct and complete information provision, which is also aligned with the specific needs of the family. - The norm is that the clinicians are not involved with the patients on waiting lists for organ and/or tissue transplants.   In the case of “(presumed) consent” donor registrations:   - Asking for family consent is no longer necessary. - Clinicians can explain how the (presumed) consent registration was established. - As clinicians should act according to patients’ wishes and the donor registration is leading, they therefore should make an effort to pursue the donation. - In addition to oppositions when the donor registration is not in line with the wishes of the patient, families can disapprove of the donation itself if they would experience psychological damage from the donation. Clinicians try to explore and meet families’ needs, values and difficulties; they also need to stress patients’ registered consent for donation. Providing correct and complete information might mitigate some family resistance. Friends or acquaintances cannot submit oppositions to donation, as only first- and second-degree family members are allowed to do so. - The clinician has the final and professional judgement of whether to pursue the donation or to make the registration invalid by being convinced about family opposition.   In the case of “family consent” or “consent of a specific person”:   - Asking for consent is necessary in addition to informing the family or specific person about the potential donation. A hierarchy of family members who are allowed to decide is determined by law. - At the end of the conversation, the clinician should summarise whether consent was given and inform the family that consent can be withdrawn at any time. - Donation is not pursued when no consensus can be reached amongst the family. - Notably, some patients might still have no registration in the opt-out system, such as patients who have recently changed their donor registrations in writing, have just turned 18 years old or have lived in the Netherlands for less than three years. In these cases, family consent is also formally necessary.   In the case of “refusal of donation” or “refusal of certain organs or tissues”:   - Patients’ refusals cannot be overruled by the family.   More information (in Dutch): <https://www.transplantatiestichting.nl/medisch-professionals/kwaliteitsstandaard-donatie> and <https://www.rijksoverheid.nl/onderwerpen/orgaandonatie-en-weefseldonatie/actieve-donorregistratie> |
| --- |

**Additional file 2.** Topic list for clinician interviews.

| *An interview guide was developed from the literature and discussions with the research team. Ideally, nondirective, open-ended questions were asked. Questions were adjusted with specific case information, as all interviews applied to one specific case. Direct observations and audio recordings were used to inform the researcher about which elements to emphasise in the interviews.* *Notes were made during and after each interview.*  **1) Before the donor conversation**  *This part of the interview was used to gain information on the case and participants in the donor conversation and to establish an interviewer-interviewee relationship.*   - Case information - Expectations and familiarity with the family - Prior experience of clinician - Preparation   **2) During the donor conversation**  *The interview mainly focused on this part.*   - Patient’s donor registration and implications for the conversation - Roles of the participants (clinician[s], nurse and family member[s]) - Information provision - Donation decision and, if applicable, resolving differences and credible oppositions - New donor law and the “Kwaliteitsstandaard Donatie” (Quality Standard for Donation [QSD]) and how these are used. The researcher asks how specific concepts in the QSD (e.g., “dialogue”, “consensus”, “proper treatment”, “professional judgement”) are applied. - Family concerns   **3) After the donor conversation**   - Aftercare regarding donation - Comparison with other cases - Final reflection - Personal opinion on donation and the new donor law |
| --- |

**Additional file 3.** Coding scheme for (presumed) consent cases and supplementary interviews with clinicians.^1^

**Clinicians’ points of departure for a donor conversation**

Clinicians’ personal considerations and preferences

- Own opinions about donation
- Own opinions about the concluded course of action in the donor conversation
- Clinicians’ resistance against (part of) the new donor law
- Balancing three perspectives continuously

- Patient (donor) vs. donor recipient

- Family vs. donor recipient

- Patient (donor) vs. family

- Continuous guiding of the family throughout the donor conversation and the donation process
- Goals of the donor conversation

- Abide by the new donor law

- Fulfil patient’s donation wish

- Reaching consensus with the family and avoiding conflicts

- Enable optimal grieving for the family

- Retrieve organs and tissues for (anonymous) recipients

Clinicians’ prior experiences with the family that made introducing donation feel more comfortable for clinicians

- Clinicians’ expectations

- Anticipate expected family resistance to donation

- Openly starting the conversation, without or despite expectations

- Expecting that the donor conversation will be straightforward or easy

- Regular and/or pleasant prior contact moments between clinician and family
- Good mutual relations within family
- Families’ prior awareness or agreement with donor registration
- Families’ prior knowledge about donation and the new donor law with its implications
- Receptiveness of the family to discuss donation: some measure of calmness is required

- Clinicians’ judgement of the complexity of the case

Contextual factors in clinicians’ professions

- Knowledge about and experience with donor conversations in general and with the new donor law in particular
- Preparation for the donor conversation
- Value of colleague support in the donor conversation

- Donation is a team effort (e.g., with intensive care unit nurses and organ donor coordinators)

- Discuss with colleagues how to pursue the conversation when the family expresses difficulties with the donation

- Daily practice at the Intensive Care Unit

- Finite time to discuss donation

**Routes of discussing patients’ donor registrations in donor conversations**

Consent cases

*Route A: Consent*

- Consent is a clear donation wish
- Mental competency of the patient is assumed
- Statement and explanation of consent registration and waiting for a response of the family
- Wishes of the patient are central

- Family opposition in the case of a “consent” registration requires more well founded reasons than in the case of a “presumed consent” registration

- Family has a limited role; limited family involvement

- Brief family verification of patients’ wishes

- Family awareness of donor registration and its implications

- Family opinions

- Implicit family reactions are sufficient

- After the conclusion of donation

- Exclusive attention to family grief processing

- Providing the family with a sense of control

- Certain choices in donation procedures

- Often no tissue donation when organs are donated

- Challenging for families to discontinue a donation procedure after confirmation and initiation

Presumed consent cases

- Statement and explanation of presumed consent registration

- Concerns whether presumed consent is an actual donation wish

- Feeling concerned about introducing and presuming donation

- Expecting family resistance

- Assumption that presumed consent is a conscious donation wish made by a mentally competent patient

- Mental competency of patient is questionable
- Determining the reason why the patient is registered with presumed consent
- Family verification of patients’ wishes and their mental competence
- Families should themselves express their doubts
- “The law” as conversation starter

- Clinician is the messenger

- Emphasising clinicians’ impartiality

- Collectively going through the donation process (clinicians and family)

- Support to introduce the topic of donation

- By the new donor law

- By emphasising potential positive donation results

*Route B: Presumed consent*

- Presumed consent is a conscious donation wish made by a mentally competent patient
- The wish of the patient is central, and family has a limited role

- Donor conversations are not shared decision-making conversations

- Support by the new donor law in the donor conversation
- Ways to cope with families who challenge or question aspects of the donation

- Emphasising the donation wish of the patient

- Emphasising implications of the new donor law

- Emphasising support of the clinician or the hospital of the implications of the law

- Emphasising the potential positive results for donor recipients and the efficiency of the national system of organ donation

- Making an effort to effectuate the donation

*Route C: Consensus*

- Clinicians want to involve the family

- No clear conclusion (initially) about patients’ wishes

- Giving the family a sense of participation and concluding that a course of action is inevitable

- Reaching consensus and avoiding conflicts with the family

- Preventing breaches of trust and complaints

- Communicating the donation with caution
- Ways to cope with family resistance or questions about donation

- Monitoring and guiding resistance is essential

- Exploring family resistance and asking questions (who and what)

- Providing information about donation

- Giving more time to discuss donation amongst family (without the clinician’s presence)

- Anticipating family structures and dynamics

- Weighing the pros and cons of donation

- Creating a safe environment, emphasising good contact between clinician and family

- Some steering/framing to donation is allowed

- Oppositions more easily approved than in the case of consent

- Initial family resistance is difficult to reverse

- Persuasion is unwanted and often considered counterproductive

- Valid reasons for not pursuing donation

- The clinician decides regarding the validity of the reasons for nondonation

- Not the known wish of the patient

- Harm to the grieving process of the family

- Conflict with religion

- Doubts about patients’ mental capacities

- Conflicts with the family or complaints

- After the conclusion for donation

- Possibility of withdrawing from the initiated donation procedure

*Route D: Family consent*

- The family decides about donation
- The complexity of the case does not suit the new donor law
- Grieving families are emphasised compared to the donor registration of the patient

- Giving time to process the situation

- Emphasising family guidance

- Emphasising positive aspects of the donation procedure, e.g., longer time with their relatives [the potential donors]

- Clinicians concluding a course of action themselves

- Language expressions suggesting family decisional capacity
- Additional consent sometimes needed for tissue donation

^1^Open codes are available on request. Not all results in the coding scheme are elaborated in the Results section. The routes were not mutually exclusive, and clinicians varied between routes during the conversations. For example, elements of family involvement (Route C) were also present in Routes A, B, or D. Moreover, the routes were not linear processes in time: elements within the routes occurred at different times in the conversations. Here, our aim is to merely present the routes that clinicians applied in response to patients’ donor registrations. These do not present causality of whether a route results in donation or not. Therefore, such conclusions cannot be drawn based on these routes.

**Additional file 4.** Consolidated criteria for reporting qualitative studies (COREQ): 32-item checklist.

Developed from:

Tong A, Sainsbury P, Craig J. Consolidated criteria for reporting qualitative research (COREQ): a 32-item checklist for interviews and focus groups. Int J for Qual Health Care 2007;19(6):349-57.

| **Item number** | **Guide questions/description** | **Reported in section** |
| --- | --- | --- |
| **Domain 1: Research team and reﬂexivity** | | |
| *Personal Characteristics* | | |
| 1. Interviewer/facilitator | Which author/s conducted the interview or focus group? | Contributions on Unblinded title page |
| 2. Credentials | What were the researcher’s credentials? E.g., Ph.D., M.D. | Untitled title page |
| 3. Occupation | What was their occupation at the time of the study? | Untitled title page |
| 4. Gender | Was the researcher male or female? | Untitled title page (name) |
| 5. Experience and training | What experience or training did the researcher have? | SO is a qualitative researcher |
| *Relationship with participants* | | |
| 6. Relationship established | Was a relationship established prior to study commencement? | N/A |
| 7. Participant knowledge of the interviewer | What did the participants know about the researcher? E.g., personal goals, reasons for conducting the research | Information letter and consent form |
| 8. Interviewer characteristics | What characteristics were reported about the interviewer/facilitator? E.g., bias, assumptions, reasons and interests in the research topic | N/A |
| **Domain 2: Study design** | | |
| *Theoretical framework* | | |
| 9. Methodological orientation and Theory | What methodological orientation was stated to underpin the study? E.g., grounded theory, discourse analysis, ethnography, phenomenology, content analysis | Methods, Data analysis |
| *Participant selection* | | |
| 10. Sampling | How were participants selected? E.g., purposive, convenience, consecutive, snowball | Methods, Case inclusion. |
| 11. Method of approach | How were participants approached? E.g., face-to-face, telephone, mail, email | WFA and SO called the donation intensivists* of the participating hospitals. These donation intensivists and the researcher informed all clinicians verbally and in writing about the study. |
| 12. Sample size | How many participants were in the study? | Results, first paragraph |
| 13. Nonparticipation | How many people refused to participate or dropped out? Reasons? | Results, first paragraph |
| *Setting* | | |
| 14. Setting of data collection | Where was the data collected? E.g., home, clinic, workplace | Methods, Design and setting and Data collection |
| 15. Presence of nonparticipants | Was anyone else present besides the participants and researchers? | Results, Table 2 |
| 16. Description of sample | What are the important characteristics of the sample? E.g., demographic data, date | Results, first paragraph, Table 2, and Additional file 5 |
| *Data collection* | | |
| 17. Interview guide | Were questions, prompts, guides provided by the authors? Were they pilot tested? | Methods, Data collection, and Additional file 2 |
| 18. Repeat interviews | Were repeat interviews carried out? If yes, how many? | N/A |
| 19. Audio/visual recording | Did the research use audio or visual recording to collect the data? | Methods, Data collection |
| 20. Field notes | Were ﬁeld notes made during and/or after the interview or focus group? | Methods, Data collection, and Additional file 2 |
| 21. Duration | What was the duration of the interviews or focus group? | Results, first paragraph, and Additional file 5 |
| 22. Data saturation | Was data saturation discussed? | Methods, Data Analysis |
| 23. Transcripts returned | Were transcripts returned to participants for comment and/or correction? | Methods, Data collection |
| **Domain 3: Analysis and ﬁndings** | | |
| *Data analysis* | | |
| 24. Number of data coders | How many data coders coded the data? | Figure 1 |
| 25. Description of the coding tree | Did authors provide a description of the coding tree? | Additional file 3 |
| 26. Derivation of themes | Were themes identiﬁed in advance or derived from the data? | Methods, Data analysis, and Figure 1 |
| 27. Software | What software, if applicable, was used to manage the data? | Methods, Data analysis |
| 28. Participant checking | Did participants provide feedback on the ﬁndings? | Methods, Data collection, and Figure 1 |
| *Reporting* | | |
| 29. Quotations presented | Were participant quotations presented to illustrate the themes/ﬁndings? Was each quotation identiﬁed? E.g., participant number | Results, description of the themes, and Table 3 |
| 30. Consistency of data and ﬁndings | Was there consistency between the data presented and the ﬁndings? | Results and Table 3 |
| 31. Clarity of major themes | Were major themes clearly presented in the ﬁndings? | Results, description of the themes |
| 32. Clarity of minor themes | Is there a description of diverse cases or discussion of minor themes? | Results, description of the themes |

*A donation intensivist is an intensivist with a specific focus on donation.

**Additional file 5.** Characteristics of cases of organ and tissue donor conversations (n=15), patients (n=15) and clinicians (n=16) in an opt-out system.

| Characteristic | Number (%) | Median (range) |
| --- | --- | --- |
| Family donor conversations (n=15) | | |
| Moment during the day  Morning  Afternoon  Evening  Night | 3 (20)  7 (46.7)  2 (13.3)  3 (20) | **-** |
| Decoupled, yes^a^ | 10 (66.7) | **-** |
| Duration of donor conversation(s) (minutes in total)^b^ | **-** | 19 (10-55) |
| Number of attendees  Health care professionals and researcher  Family members and friends of the patient | **-** | 3 (2-5)  3 (2-7) |
| Type of donation – discussed^c^  Donation after Circulatory Death (DCD)  Donation after Brain Death (DBD)  Tissue donation | 12 (80)  11 (73.3)  8 (53.3) | **-** |
| Patients (n=15) | | |
| Gender, female | 5 (33.3) | - |
| Age (years) | - | 61 (25-72) |
| Critical injury  Cardiac Arrest  Cerebral Vascular Accident  Intracranial Haemorrhage^d^  Myocardial Infarction  Not known (Pulmonary Embolism)  Traumatic Brain Injury^e^ | 1 (6.7)  2 (13.3)  7 (46.7)  1 (6.7)  1 (6.7)  3 (20) | - |
| Length of stay (days) | - | 1 (0.1-14) |
| Donor registration  Consent  Presumed consent | 7 (46.7)  8 (53.3) | - |
| Donation - result  Initiated and successful procedure^f^  DCD  DBD  Initiated procedure, but no successful procedure^g^  No initiated procedure due to family opposition | 10 (66.7)  7  3  3 (20)  2 (13.3) | - |
| Clinicians (n=16) | | |
| Gender, female | 5 (31.3) | - |
| Age (years) | - | 42 (30-62) |
| Medical profession  Donation intensivist^h^  Fellow  ICU resident not in training  Intensivist | 4 (25)  5 (31.3)  1 (6.3)  6 (37.5) | - |
| Experience (years)  At the Intensive Care Unit  In the field of organ and tissue donation | - | 11 (1-31)  11 (2-31) |
| Familiarity with Quality Standard for Donation  Theoretical training^i^  Practical training | 8 (50)  1 (6.3) | - |
| Personal donor registration  No consent  Consent  Presumed consent  Family consent  No registration | 1 (6.3)  12 (75)  1 (6.3)  1 (6.3)  1 (6.3) | - |
| Timing of interview after the donor conversation (days) | - | 19 (7-45) |
| Type of interview  Face-to-face in the work environment  Telephone  Video | 1 (6.3)  3 (18.8)  12 (75) | - |
| Duration interview (minutes) | - | 52 (31-61) |

^a^From the bad news conversation.

^b^Rounded to minutes. When the donor conversation is not decoupled from the bad news conversation, the duration includes the duration of the bad news conversation and the donor conversation together. The donor conversations of cases 8 and 10 were separated into two conversations.

^c^Various types of donations could be discussed in the conversations.

^d^One case also had secondary cardiac arrest.

^e^With or without secondary hypoxic brain injury.

^f^Tissues were also donated in two of these cases (one DBD case and one DCD case).

^g^Initiated procedure, but no donation because organs and, if applicable, tissues were not usable/rejected, or

the patient did not die within two hours (for DCD).

^h^A donation intensivist is an intensivist with a specific focus on donation.

^i^Only three clinicians completed the theoretical training, including three e-learning modules.

**REFERENCES**

1. Reinders MEJ, Reiger-van de Wijdeven JMMPJ, de Jonge J, et al. Dutch Law Approves Opt-out System. Transplantation. 2018;102(8).

2. Werkgroep Ontwikkeling Kwaliteitsstandaard NTS. Kwaliteitsstandaard Donatie 2020. Available from: https://www.transplantatiestichting.nl/files/2020-12/nts-kwaliteitsstandaard-donatie-versie1.1-april-2020.pdf?c5f7988255 (accessed 20-4-2022).

3. Nederlandse Transplantatie Stichting. Modelprotocol Postmortale orgaan- en weefseldonatie 2022. Available from: https://www.transplantatiestichting.nl/files/2022-02/modelprotocol-versie-5-februari-2022.pdf (accessed 20-4-2022).

4. Sheldon T. Dutch to start presumed consent for organ donation in 2020. BMJ. 2018;360:k768.

5. Wet op de orgaandonatie: Volksgezondheid, Welzijn en Sport (2022). Available from: https://wetten.overheid.nl/BWBR0008066/2022-01-01/ (accessed 28-09-2022).

6. Nooren. Gewijzigde motie-Nooren (PvdA) c.s. over het opstellen van een kwaliteitsstandaard voor transplantatiezorg 2018. Available from: https://www.eerstekamer.nl/motiedossier/33506_s_gewijzigde_motie_nooren (accessed 13-12-2022).

7. Jansen NE, Williment C, Haase-Kromwijk BJJM, et al. Changing to an Opt Out System for Organ Donation—Reflections From England and Netherlands. Transpl Int. 2022;35.
